# Epithelial‐Mesenchymal Transition in colorectal cancer: Annexin A2 is caught in the crosshairs

**DOI:** 10.1111/jcmm.16962

**Published:** 2021-10-08

**Authors:** Murilo Ramos ROCHA, Jose Andres Morgado‐Diaz

**Affiliations:** ^1^ Grupo de Estrutura e Dinâmica Celular Programa de Oncobiologia Celular e Molecular Instituto Nacional de Câncer Rio de Janeiro Brasil

**Keywords:** annexin A2, cofilin 1, colorectal cancer, epithelial‐mesenchymal transition

## CONFLICTS OF INTEREST

The authors declare no conflict of interest.

## AUTHOR CONTRIBUTIONS

**Murilo Ramos Rocha:** Conceptualization (equal); Formal analysis (equal); Investigation (equal); Writing‐original draft (lead); Writing‐review & editing (equal). **Jose Andres Morgado‐Diaz:** Conceptualization (equal); Data curation (equal); Formal analysis (equal); Funding acquisition (lead); Investigation (equal); Writing‐original draft (equal); Writing‐review & editing (equal).

To the Editor,

Epithelial‐mesenchymal transition (EMT) is a complex cellular program where cells transit between epithelial and mesenchymal phenotypes. It is mainly characterized by the loss of apical‐basal polarity, disassembly or reorganization of cell‐cell junctions and cytoskeleton. Epithelial features are lost in favour of mesenchymal ones, increasing motility and invasiveness.^1^ However, EMT’s role in the metastatic cascade has been controversial. Innumerous articles indicate EMT involvement in basal membrane rupture, intravasation, resistance to the shear stress in blood vessels and extravasation,^2^, ^3^, ^4^ but some researchers have already shown that EMT was not essential for metastatic colonization.^5^, ^6^ This might be explained by the multiplicity of possible outcomes for a cell undergoing EMT. Pastushenko and collaborators showed that there are several intermediate stages in this process that contribute to the formation of subpopulations that differ in proliferation, invasion, plasticity and metastatic capabilities.^7^ This plasticity allows cells to undergo reversible changes between epithelial and mesenchymal features adapting to diverse hostile conditions.^2^ These properties make EMT‐related proteins interesting markers and/or therapeutic targets to prevent metastasis.

Fuelled by transcriptomic data sets, several groups created molecular subclassifications of specific types of cancers to guide therapeutic strategies. For colorectal cancer (CRC), this effort was tackled by six groups that coalesced their individual subclassifications into one consensus molecular subtype (CMS) index. Taking into consideration mRNA expression, morphological and clinical information, four subtypes were proposed: CMS1 (Immune), CMS2 (Canonical), CMS3 (Metabolic) and CMS4 (Mesenchymal).^8^ Comprising 23% of the analysed patients, CMS4 is characterized by EMT markers, strong stromal activation, angiogenesis and the worst prognosis among the subtypes.

Puzzled by this scenario, we investigated whether Annexin A2 (ANXA2), a phospholipid‐binding protein, already described to be associated with EMT in pancreatic ductal carcinoma,^9^ non‐small cell lung cancer^10^ and breast cancer,^11^ was associated with CRC progression. We observed that ANXA2 is overexpressed in CRC patients, especially in stage IV tumours or metastasis,^12^ which strengthens the observations of Yang et al.^13^ that proposed ANXA2 as a diagnostic and prognostic marker for CRC in a cohort of 150 patients. Analysis of The Cancer Genome Atlas Colon Adenocarcinoma (TCGA‐COAD) and Rectal Adenocarcinoma (TCGA‐READ) databanks showed that ANXA2 transcript is overexpressed in all stages of tumoural progression and is differentially expressed between CMSs. Surprisingly, CMS1 had the highest expression of ANXA2, not CMS4 which is characterized with the classical markers of EMT.^12^ Possible explanations for this association lie in the role of ANXA2 heterotetramer in immune response via plasmin activation^14^ and in the relation between ANXA2 and STAT activation.^12^, ^15^, ^16^, ^17^, ^18^ Also, the recently described association of ANXA2 with the immune microenvironment in hepatocellular carcinoma, might give us another clue to why.^19^

Unlike previous works that used EGF, HGF or IGF‐1 to induce EMT and evaluate ANXA2 expression,^9^, ^10^, ^11^ our TGF‐ß treatment led to elevated ANXA2 protein levels, elongated cellular morphology, E‐cadherin internalization and vimentin upregulation. ANXA2 silencing was able to prevent invasiveness due to TGF‐ß and inhibitors of the Src/ANXA2/STAT3 pathway prevented EMT. It was also noticeable that ANXA2 was colocalized with the internalized E‐cadherin.^12^ There is evidence that ANXA2 participates in the physiological regulation of cellular junctions. Yamada et al.^20^ suggested a role in adherens junction (AJ) formation considering that ANXA2‐silenced MDCK cells were not able to re‐establish AJ during a calcium switch assay. Our finding was re‐enforced by those of Chen et al.^21^ that also found a relation between TGF‐ß activation and ANXA2‐mediated E‐cadherin internalization during the EMT. ANXA2 might also control TGF‐ß induction of EMT regulating c‐Myc mRNA translation.^22^, ^23^

Chojnacka et al.^24^ described ANXA2 role in maintaining the integrity of the blood‐testis barrier through the regulation of the cytoskeleton. ANXA2 knockdown changed the localization of multiple proteins of the apical junctional complex and actin regulators (Arp3, cortactin and dynamin I/II) causing the loss of the barrier functionality. Overexpression of ANXA2 can also lead to cytoskeleton reorganization. Being capable to interact directly with F‐actin through its C‐terminal portion,^25^ ANXA2 promotes tumour progression through motility structures remodelling^26^ or regulation of the endocytic trafficking.^27^ In its heterotetrameric form with S100 proteins, ANXA2 exerts bundling activity over actin filaments.^28^ Monomeric ANXA2 also caps and regulates the barbed ends of growing actin filaments interacting directly with globular actin units.^29^ Using phospho‐mutated isoforms of ANXA2, de Graauw et al.^30^ unravelled a downstream mechanism of actin modulation through LIMK activation and posterior phosphorylation of cofilin‐1 (CFL1). ANXA2 knockdown in MDA‐MB‐231 cells resulted in CFL1 dephosphorylation and enhanced EGFR endocytosis. The effect of ANXA2 over EGFR endocytosis through CFL1 was confirmed with the transfection of a CFL1 phospho‐mimicking mutant that restored endocytosis in shANXA2 cells.^27^

Responsible for severing filamentous actin into globular monomers, CFL1 controls the actin treadmilling and thus drives membrane protrusion, cell migration and invasion. Studies have demonstrated that the RhoA‐ROCK‐LIMK pathway, responsible for CFL1 regulation, plays a pivotal role in the disassembly of junctional complexes by remodelling cortical actin and causing E‐cadherin redistribution in the cell.^31^ Even though CFL1 is found overexpressed in several tumours, its activation status will be the determinant in cytoskeleton rearrangement and tumoural progression.^32^ CFL1 activity is regulated by LIMK1/2 proteins, its activity is inhibited by phosphorylation at serine 3, hindering its ability to bind and sever F‐actin, and by phosphatase slingshot homolog 1 (SSH1) that dephosphorylates and activates CFL1.^33^ To assess the role of p‐CFL1, we evaluated EMT parameters in CFL1 S3E phospho‐mimetic mutant. This mutant was able to recapitulate EMT‐associated changes without TGF‐ß treatment: reduced E‐cadherin and claudin‐3 in cellular contacts and higher vimentin protein levels.^34^ These findings suggest that CFL1 is crucial in the EMT, cell migration and invasion by regulating actin cytoskeleton organization and impacting the apical junctional organization during the EMT.

**FIGURE 1:**
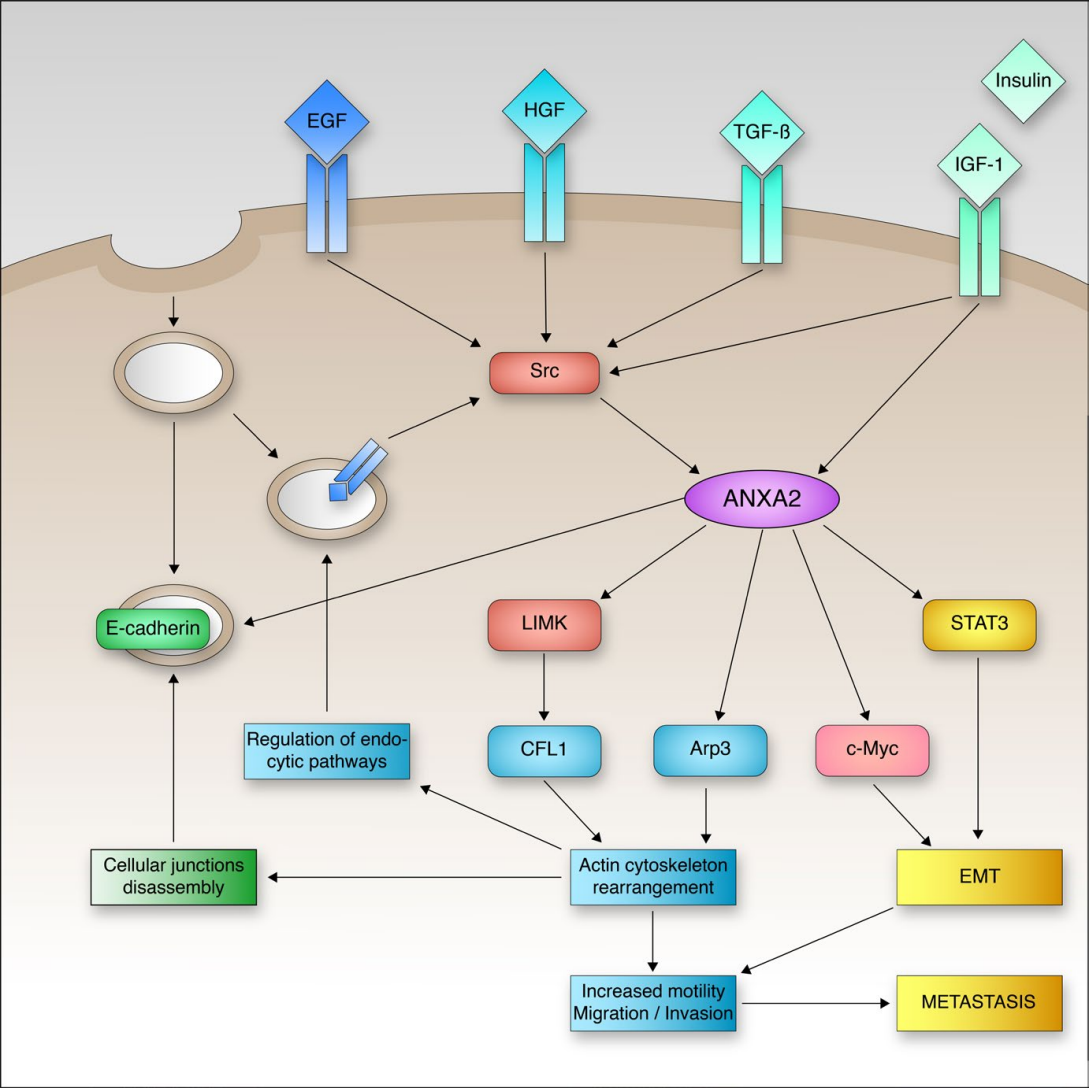
Overview of ANXA2 proposed pathway in EMT‐related processes. Through its phosphorylation after growth factor stimuli, ANXA2 can lead to LIMK phosphorylation and CFL1 inactivation. This pathway can cause the rearrangement of actin cytoskeleton and regulate endocytic pathways and the disassembly of cellular junctions. Phosphorylated ANXA2 may also provoke STAT3 phosphorylation, which will translocate to the nucleus and induce the EMT transcriptional program. These changes combined originate more motile cancer cells that will favour metastasis formation. ANXA2 control over c‐Myc mRNA translation also has an impact on EMT induction after growth factor stimuli

The multiplicity of intermediate stages and cellular functions comprised in the EMT program makes it a difficult but highly promising target for cancer therapy. Here, we pointed out two proteins involved with its induction: ANXA2 and a downstream effector CFL1. Acting directly on actin filaments to promote cytoskeleton reorganization, CFL1 lies downstream to several pathways involved in the EMT program. However, its direct involvement with multiple physiological functions makes it hard to target without disturbing the cellular milieu. ANXA2, on the other hand, is already a promising target for cancer treatment. Overexpressed in several tumour types, ANXA2 not only lies upstream of CFL1 in its regulation pathway but also represents a convergence point of multiple growth factor‐activated pathways. Growth factors like HGF, EGF, IGF‐1, insulin and TGF‐ß can phosphorylate ANXA2 in its tyrosine 23 residue through Src kinase or direct activation. Once phosphorylated, ANXA2 promotes a wide of range of cellular changes leading to tumour progression (Figure 1). There are multiple research groups currently looking for ways to therapeutically interfere with ANXA2. Dr. Choo's lab in Singapore developed a novel monoclonal antibody ‘ch2488’ that targets a unique glycan epitope of ANXA2. This antibody was able to impair ovarian and breast cancer progression and now it is been tested in a CAR‐T therapy against ovarian cancer.^35^ Sharma et al.^36^ published pre‐clinical results for anti‐ANXA2 use against triple‐negative breast cancer. Staquicini et al.^37^ described a new peptide motif that recognizes and inhibits intracellular ANXA2, impairing tumour cell adhesion, migration and in vivo grafting. Dr Pasqualini has also described a peptide that recognizes extracellular ANXA2^38^ that may be used for a more targeted strategy. Dr Zheng's group from Johns Hopkins University, after establishing ANXA2 as a valuable pancreatic ductal adenocarcinoma target, developed a *Listeria*‐based GVAX vaccine anti‐ANXA2 that generates a T‐cell tumour antigen‐specific response and sensitizes PDAC to checkpoint inhibitor therapy.^39^ There is still much to learn regarding ANXA2 and the pathways orchestrated by it; its involvement in pre‐metastatic niche preparation through extracellular vesicles, role in chemo and radioresistance; and these just add to the expectations of promising new therapeutic approaches. ANXA2 has also been used by researchers with distinct purposes such as the development of peptides that recognize its phosphorylated form and allow precise fluorescence‐guided surgical tumour resection.^40^

Despite all attempts to use ANXA2 as a target in anti‐cancer treatment, not much has been done regarding CRC. We highlighted here ANXA2 involvement in the EMT process of CRC cells through direct cellular junction regulation, cytoskeleton remodelling and activation of growth factor signalling pathways (Figure 1). These functions, among others performed by ANXA2, place it in the crosshairs of CRC researchers as a viable target for novel directed treatments. It is important to highlight that being a pleiotropic protein, the understanding of the importance of each protein region through site‐specific mutational studies might provide opportunities to target ANXA2 without unintended effects.

## Data Availability

Data sharing is not applicable to this article as no data sets were generated or analysed during the current study.
